# Association Between Calculated Small Dense Low-Density Lipoprotein Cholesterol (sdLDL-C) and Soft Carotid Plaques on CT Angiogram of the Head and Neck

**DOI:** 10.7759/cureus.65292

**Published:** 2024-07-24

**Authors:** Thulasi Rajan, Ganesh M, Sowmya K, Easwar L

**Affiliations:** 1 Biochemistry, Sri Ramachandra Institute of Higher Education and Research, Chennai, IND; 2 Radiodiagnosis, Sri Ramachandra Institute of Higher Education and Research, Chennai, IND

**Keywords:** small dense low-density lipoprotein, cerebrovascular accident (stroke), low-density lipoprotein cholesterol (ldl-c), ct angiogram, sdldl, carotid plaque

## Abstract

Background: Cerebrovascular accident (CVA), also commonly known as stroke, is an acute condition characterized by jeopardized perfusion of the brain tissue. Atherosclerosis is a common converging point for the various risk factors for CVA. It is a chronic, evolving condition of the vessel wall characterized by peculiar lesions known as atheromas. Low-density lipoprotein cholesterol (LDL-C) has been one of the established and traditional risk factors for the development of plaques in atherosclerosis. Small dense LDL-C (sdLDL-C) is a subclass of LDL-C that is considered more atherogenic, and its role in atherosclerotic plaque formation has been very well established. Hence, in this study, we aimed to find the association between calculated sdLDL-C and atherosclerotic carotid plaque (including various plaque characteristics).

Materials and methods: This retrospective cross-sectional study was conducted at Sri Ramachandra Medical College and Research Institute between December 2022 and December 2023 after getting ethics approval from the Institutional Ethics Committee. Patients who underwent CT angiogram (312) were included in the study, and their lipid profile data were collected from the Laboratory Information System. Participants were divided into groups depending on the presence or absence of carotid plaque, the characteristics of the plaque, and the narrowing caused by the plaque. sdLDL-C was calculated using Sampson formula from the lipid parameters in these groups. Statistical analysis was done using SPSS Statistics version 16.0 (SPSS Inc. Released 2007. SPSS for Windows, Version 16.0. Chicago, SPSS Inc.). A p-value of <0.05 was considered significant.

Results: sdLDL-C was significantly higher in the plaque group (37.25 ± 13.69 mg/dL) when compared to the group without plaques on CT angiogram (34.09 ± 11.64 mg/dL) (p<0.05), wherein the LDL-C wasn’t significantly different between the two groups. sdLDL-C was also elevated in the soft plaque sub-group (39.46 ± 13.63 mg/dL) when compared to the calcific plaque sub-group (35.41 ± 13.05 mg/dL), which was statistically significant (p<0.05).

Conclusion: sdLDL-C is associated with atherosclerotic carotid plaques, especially the soft plaques on CT angiogram, which are considered to be vulnerable plaques. Thus, calculated sdLDL-C can be utilized as a cost-effective tool to assess plaque vulnerability and monitor hypolipidemic treatment in addition to LDL-C.

## Introduction

Cerebrovascular accident (CVA), also commonly known as stroke, is an acute condition characterized by jeopardized perfusion of the brain tissue. Among all CVAs, around 85% are ischemic, caused by an emboli or thrombi, with the rest being hemorrhagic [1]. Stroke continues to be the second-leading cause of mortality globally and the third-leading cause of mortality and disability combined [2]. The risk factors for CVA include hypertension, dyslipidemia, diabetes mellitus, smoking, and others. Atherosclerosis, which is the one common converging factor for these risk factors, constitutes a substantial portion of large and small vessel disease, which is considered a significant cause of ischemic stroke [3].

Atherosclerosis is a chronic, evolving condition of the vessel wall characterized by peculiar lesions known as atheromas. The evolution starts with a diffuse thickening of the tunica intima and its progression to a pathological thickening (with a more necrotic lipid core), followed by the development of fibroatheroma. This progression of the benign lesion into a more pathological lesion is determined and controlled by poor circulatory flow mechanics and the lesion’s increased affinity for the circulating lipoproteins, especially low-density lipoprotein cholesterol (LDL-C) [4,5].

LDL-C has been one of the established and traditional risk factors for the development of plaques in atherosclerosis. It is still considered one of the primary targets of hypolipidemic treatment in the National Cholesterol Education Program Adult Treatment Panel (NCEP ATP). Yet, atherosclerotic plaques continue to appear even in individuals with well-controlled lipid parameters [5]. Research studies have shown that the LDL-C is an entity covering various subclasses (I, II, III, and IV) rather than a single lipoprotein molecule. These subclasses differ in their properties, such as density, size, shape, and even in their potential to cause atherosclerosis. Various analytical techniques have been employed to separate the subclasses, among which analytical centrifugation (AC), gradient gel electrophoresis (GGE), and nuclear magnetic resonance spectroscopy are the most frequently used. The LDL subclasses III and IV, as measured by the AC and GGE, comprise the small dense LDL-C (sdLDL-C), which is considered to be more atherogenic than its counterpart, the large buoyant LDL-C (LDL sub-classes I and II) [6].

sdLDL-C and its involvement in pathological processes have been studied in various disorders, and its role in atherosclerotic plaque formation has been very well established. It is hypothesized that the production of sdLDL-C from the liver is dependent on its endogenous triglyceride content (higher triglyceride content leads to increased production of sdLDL-C). The factors that make sdLDL-C more atherogenic include (i) its content, which modifies the interaction with the Apo B-100 apolipoprotein and sequentially delays its uptake into the liver through the LDL receptor. This leads to its increased circulating time [7]. Other factors include (ii) the small size, which makes it easy to penetrate the endothelium [8]. sdLDL-C also has a poor intrinsic anti-oxidative mechanism, making it susceptible to more oxidative stress and modifications like desialylation and glycation, rendering it more electronegative and helping it to adhere to the proteoglycans of the vessel wall [6]. Increased circulating time, with oxidative modifications, makes these particles a menace to the endothelium and the sub-endothelial space, leading to the development of atherosclerotic plaques.

CT angiogram of large vessels has been routinely used as an initial diagnostic tool in CVA cases. The most common cause of ischemic stroke is thromboembolism because of a ruptured atherosclerotic plaque of a large artery (the carotid, the middle cerebral, and the vertebral arteries) and cardiac diseases [9].

In this study, we try to find out the relationship between the carotid plaque characteristics, such as the nature of the plaque, the severity of luminal narrowing as seen on CT angiogram, and the calculated sdLDL-C.

## Materials and methods

This retrospective cross-sectional study was conducted at Sri Ramachandra Medical College and Research Institute from December 2022 to December 2023 (one year). The study was conducted after getting ethics approval from the Institutional Ethics Committee of Sri Ramachandra Institute of Higher Education and Research (approval number: CSP-MED/24/JAN/97/15). The patients who underwent CT angiogram of the head and neck for various indications, including CVA, transient ischemic attacks, and dizziness, were included in the study (450 participants). Participants who didn’t have lipid profile data were excluded from the study (138 participants). Three hundred and twelve participants were divided into two groups based on the presence or absence of atherosclerotic carotid plaques: in the internal carotid artery or in the common carotid artery. Lipid profile data, which included total cholesterol, triglycerides, LDL-C, and high-density lipoprotein cholesterol (HDL-C), were collected from the Laboratory Information System. (The samples of the participants were analyzed by the Beckman Coulter AU 680 Clinical Chemistry Analyzer, Beckman Coulter Inc., Brea, CA, USA, using enzymatic assays.) The sdLDL-C was calculated from the lipid parameters using Sampson formula. The sdLDL-C that was calculated using this formula had a strong correlation with the homogenous sdLDL-C assay described by Ito et al. [10].

CT angiogram was performed using the 128-slice GE optima Computed Tomography System (GE Healthcare, Chicago, IL, USA) after intravenous administration of Iohexol (Omnipaque) at 350 mg I/mL of contrast with a dosage of 1 mL/kg. Coronal and sagittal reformatted images and 3D reconstructed images were studied. The plaques noted on CT angiogram were described based on the amount of calcification present. Calcification was defined as a density of Hounsfield units (HU) higher than 130 HU. The lesions with no calcification were classified as soft plaques (40-50 HU), and those with greater than 50% calcification were classified as calcified plaques, and the remaining as mixed plaques.

CT angiogram of the head and neck reports of the participants was analyzed to identify the presence of any carotid plaque and, if present, the following characteristics: (1) the number of atherosclerotic plaques present, (2) the characteristics of the plaques that are found, and (3) the narrowing caused by the plaques that were taken into account. (As reported by two board-certified consultant radiologists with MDs in radiodiagnosis and at least eight years of experience in cardiothoracic radiology, the degree of narrowing/stenosis caused by the plaques was reported as per the North American Symptomatic Carotid Endarterectomy Trial (NASCET) criteria: stenosis was categorized as mild (<30%), moderate (30-69%), and severe (70-99%). The degree of stenosis was calculated using the formula: degree of stenosis (%) = (1- (minimal luminal diameter/diameter of normal distal ICA)) × 100)). The focus of the analysis was narrowed to the common carotid artery and the six divisions of the internal carotid artery because of their significant involvement in the formation of thromboembolism in ischemic stroke cases [9]. Also, for simplicity of analysis, the other major arteries, such as the middle cerebral artery and the vertebral artery, were not included in this study.

Carotid scoring was given based on the method mentioned by Armstrong et al. [11]. Three major segments of the carotid artery were considered, namely, the common carotid artery, the internal carotid artery, and the carotid bulb. Three segments of the carotid artery on both sides (right and left) give six segments. If a plaque was found in the designated segment, it received a score of one. A segment would only receive a score of one, even if many plaques were present. Thus, the carotid scoring ranged from one to six for a given participant, with one being the lowest and six being the highest.

Statistical analysis was done using SPSS Statistics version 16.0 (SPSS Inc. Released 2007. SPSS for Windows, Version 16.0. Chicago, SPSS Inc.). The data was checked for normality of distribution using the Shapiro-Wilks test. Continuous variables were expressed as the mean and standard deviation. Groups were divided based on (i) the presence or absence of plaques, (ii) the amount of narrowing present, and (iii) the characteristic of the plaque present. The levels of sdLDL-C and other lipid parameters were compared between these groups by a student t-test and an ANOVA test. A p-value of less than 0.05 was considered to be significant. Correlation analysis between variables was done using Pearson’s correlation.

## Results

Table 1 shows the baseline characteristics of the study population, divided into two groups based on the presence or absence of carotid plaques on CT angiogram. The patients in the plaque group had a higher mean age when compared to the other group. They also had higher mean years of diabetes (13.34 ± 7.34 vs. 7.19 ± 3.95 years) and hypertension (10.85 ± 4.06 vs. 6.42 ± 2.71 years), which was significant (p<0.05). Calculated sdLDL-C was significantly higher in the plaque group (37.25 ± 13.69 mg/dL) when compared to the group without plaques (34.09 ± 11.64 mg/dL) (p<0.05). There was no significant difference in LDL-C between these two groups.

**Table 1:** Baseline characteristics of the study population showing various parameters TC: total cholesterol, TGL: triglycerides, LDL-C: low-density lipoprotein cholesterol, HDL-C: high-density lipoprotein cholesterol, sdLDL-C: small dense low-density lipoprotein cholesterol, *: statistically significant

S. No.	Variable	Plaque group	No plaque group		P-value
1.	Age (years)	62.00 ± 11.51		46.24 ± 10.25	<0.001*
2.	DM (years)	13.34 ± 7.34		7.19 ± 3.95	<0.001*
3.	HTN (years)	10.85 ± 4.06		6.42 ± 2.71	0.006*
4.	Smoking (years)	23.63 ± 9.11		11.67 ± 2.88	0.058
5.	Alcohol (years)	18.82 ± 8.28		11.80 ± 7.82	0.133
6.	Statin medication (years)	2.56 ± 1.96		1.16 ± 0.08	0.007*
7.	TC (mg/dL)	179.67 ± 51.17		172.96 ± 47.23	0.346
8.	TGL (mg/dL)	146.73 ± 87.40		134.65 ± 67.09	0.327
9.	LDL-C (mg/dL)	117.12 ± 38.45		111.46 ± 34.68	0.288
10.	HDL-C (mg/dL)	40.64 ± 10.50		41.28 ± 10.24	0.663
11.	LDL-C calculated (mg/dL)	112.73 ± 41.48		107.60 ± 38.86	0.376
12.	sdLDL-C calculated (mg/dL)	37.25 ± 13.69		34.09 ± 11.64	0.044*
13.	Carotid score	2.95 ± 1.28		-	-
14.	Number of plaques	5.29 ± 3.01		-	-

Table 2 shows the lipid parameters across three groups classified according to luminal narrowing (i.e., mild, moderate, and severe). There was no significant difference in lipid parameters between the three groups.

**Table 2:** Comparison of various lipid parameters across three groups (classified according to the luminal narrowing) TC: total cholesterol, TGL: triglycerides, LDL-C: low-density lipoprotein cholesterol, HDL-C: high-density lipoprotein cholesterol, sdLDL-C: small dense low-density lipoprotein cholesterol

Variable	Narrowing			
	Mild	Moderate	Severe	P-value
TC (mg/dL)	182.75 ± 42.25	179.96 ± 57.30	178.88 ± 51.85	0.88
TGL (mg/dL)	145.49 ± 70.44	148.40 ± 74.76	148.81 ± 72.82	0.96
LDL-C (mg/dL)	118.71 ± 32.70	117.06 ± 41.48	117.52 ± 39.62	0.96
HDL-C (mg/dL)	42.07 ± 9.60	40.80 ± 10.45	39.18 ± 11.46	0.21
LDL-C calculated (mg/dL)	114.79 ± 35.89	112.50 ± 44.78	112.96 ± 42.41	0.93
sdLDL-C calculated (mg/dL)	37.45 ± 11.52	37.17 ± 14.91	37.75 ± 14.28	0.96

Table 3 shows Pearson’s correlation between the lipid parameters and carotid scoring. There was no significant correlation found.

**Table 3:** Correlation of lipid parameters with carotid scoring (Pearson's correlation) TC: total cholesterol, TGL: triglycerides, LDL-C: low-density lipoprotein cholesterol, HDL-C: high-density lipoprotein cholesterol, sdLDL-C: small dense low-density lipoprotein cholesterol

Variables	Carotid scoring	
	Correlation coefficient	P-value
TC (mg/dL)	0.02	0.68
TGL (mg/dL)	0.10	0.07
LDL-C (mg/dL)	0.01	0.74
HDL-C (mg/dL)	0.01	0.77
LDL-C calculated (mg/dL)	0.00	0.97
sdLDL-C calculated (mg/dL)	0.06	0.22

Table 4 shows the various lipid parameters along with calculated LDL-C and calculated sdLDL-C among three groups divided based on the characteristics of the plaque (i.e., soft, calcific, and mixed). There was no significant difference in LDL-C levels between the groups. sdLDL-C was higher in the soft group (39.46 ± 13.63 mg/dL) when compared to the calcific group (35.41 ± 13.05 mg/dL), and it was statistically significant (p<0.05).

**Table 4:** Comparison of various lipid parameters across three groups (classified as per plaque characteristics) TC: total cholesterol, TGL: triglycerides, LDL-C: low-density lipoprotein cholesterol, HDL-C: high-density lipoprotein cholesterol, sdLDL-C: small dense low-density lipoprotein cholesterol, *: statistically significant

Variable	Calcified	Soft	Mixed	P-value
TC (mg/dL)	174.24 ± 49.60	183.19 ± 51.08	192.66 ± 54.52	0.09
TGL (mg/dL)	137.62 ± 75.30	166.64 ± 70.86	150.81 ± 70.59	0.09
LDL-C (mg/dL)	112.75 ± 37.19	119.89 ± 37.79	127.57 ± 41.44	0.06
HDL-C (mg/dL)	40.73 ± 10.27	38.66 ± 10.30	42.90 ± 11.40	0.14
LDL-C(calculated) (mg/dL)	108.79 ± 40.73	114.76 ± 39.42	122.81 ± 45.39	0.13
sdLDL-C(calculated) (mg/dL)	35.41 ± 13.05	39.46 ± 13.63	40.26 ± 14.88	0.04*

## Discussion

Atherosclerosis is a disease of the vascular wall involving initial endothelial dysfunction in specific segments followed by the accumulation of lipid particles, especially LDL-C, in the tunica media. Over a period, it evolves to form an atherosclerotic plaque. This process, if unchecked, leads to debilitating complications, including cerebrovascular disease (stroke). The incidence of stroke in India has increased from 108 to 172/100,000 people per year, up from a lower rate in the 1980s. The increased incidence along with the life-threatening complications in this pathology underscores the importance of early diagnosis, monitoring, and initiation of treatment [12]. sdLDL-C is a sub-group of LDL-C that has a density ranging from 1.034-1.060 g/ml as measured by AC and a peak diameter of 22.0-25.5 nm as measured by GGE [13,14]. This specific subtype, sdLDL-C, because of its notorious characteristics, poses a persistent threat to the vessel wall in terms of initiating the atherosclerotic process.

The older concept of using luminal narrowing to identify risk has been replaced by the utilization of characteristics of the plaque such as the lipid core, fibrous cap thickness, intra-plaque hemorrhage, and smooth muscle content, and all these have been considered measures of plaque instability and determinants of plaque rupture. These features are considered superior for risk prediction in carotid atherosclerosis [15]. Considering this, CT and MR angiogram can be useful tools over traditional ultrasonography (USG), which measures the carotid artery intima-media thickness, to predict risk and monitor treatment [16]. The financial and time-consuming factors of MR angiogram make it less preferred when compared to CT angiogram. Thus, in this retrospective cross-sectional study, we aimed to compare the sdLDL-C levels between two groups, namely, patients with at least one carotid plaque on CT angiogram and patients with no carotid plaques on CT angiogram.

In baseline characteristics from Table 1, the mean age of the participants in the plaque group was significantly higher than the no-plaque group. This is consistent with the knowledge that increasing age is an established risk factor for the development of non-communicable diseases such as diabetes mellitus and hypertension and, thus, the occurrence of atherosclerotic plaques [17].

In our study, the plaque group had a significantly higher sdLDL-C level when compared to the no-plaque group (p<0.05) (Table 1). This finding is similar to a study by Li et al., who found elevated sdLDL-c in a group of patients with carotid atherosclerosis as determined by USG [18]. Another study by Ma et al., in a cohort of the Chinese population, found a significant association between sdLDL-C levels and incident carotid plaques. The sdLDL-C level was also associated with vulnerable plaque morphology, as seen by USG of the carotid arteries [19]. Another study investigated sdLDL-C in relation to carotid atherosclerosis and found a strong association between sdLDL-C levels and atherosclerotic development. The marker was also higher in the unstable plaque group when compared to the group with stable plaques [20]. sdLDL-C, because of its unique characteristics, including its smaller size, lipid content and composition, and poor anti-oxidative capacity, is more prone to oxidation, making it more vicious, thereby increasing the probability of invading the sub-endothelial space [6]. Whether oxidized sdLDL-C (which gets oxidized in the bloodstream because of its poor anti-oxidative capacity) penetrates the endothelium through various transport mechanisms or gets oxidized after entering the sub-endothelial space remains unclear [6,21]. However, sdLDL-C is considered highly atherogenic, and it must have a major role in the formation of atherosclerotic plaques.

In our study, there was no significant difference in the sdLDL-C levels between the three groups divided based on the degree of luminal narrowing caused by the plaque (Table 2). The concept of luminal narrowing was adapted mainly because of its utility in deciding on an intervention: carotid endarterectomy. However, later studies revealed that plaque characteristics that determine stability are better predictors of future ischemic strokes [22]. A study by Kamtchum-Tatuene et al. found that high-risk plaques (in terms of plaque rupture) were not associated with the severity of luminal narrowing [23]. Thus, plaque characteristics, rather than luminal narrowing, can be used to assess the risk for future adverse events.

In our study, sdLDL-C did not correlate with carotid scoring, which described the extent of the atherosclerotic disease process (Table 3). This carotid scoring reflected the number of segments of carotid arteries involved, but it did not consider the number of plaques, which reflects the plaque burden [11].

Both groups (plaque and no plaque) from Table 1 had almost similar LDL-C levels, and it could not differentiate between these two groups. This observation is reasonable because the majority of patients in the plaque group (nearly 40% of patients) were using lipid-lowering medications, which means that LDL-C levels in the normal range are to be expected [24]. About 70% of the patients in the plaque group presented with acute ischemic stroke and had LDL-C within a desirable range. It can either be because the process of plaque formation and progression would have initiated even before the start of lipid-lowering drugs and has led to this critical point of plaque rupture and subsequent thrombus formation or because a higher sdLDL-C, even with a normal LDL-C in this group, can be a factor, causing instability of the plaque and making it rupture along with its role in the genesis of the plaque. Other studies support this finding of higher sdLDL-C with a normal LDL-C level as a risk for the development of atherosclerosis [25,26].

To study the effects of lipid-lowering drugs on sdLDL levels, we studied sdLDL-C concentration in two groups, divided based on the type of lipid-lowering agent: rosuvastatin or atorvastatin. However, there was no significant difference in the mean values of sdLDL-C between the two groups. However, various other elements involved in the regulation of lipid levels, including the number of years of statin medication, compliance with therapy, diet, and other metabolic abnormalities, could also influence lipid levels [27]. A study done by Manemann et al. found that the lipid profile had significant within-individual variability, which should also be considered while commenting on these lipid parameters. They also established that a high variability in lipid profile over time has increased the risk for adverse cardiovascular outcomes [28].

In our study, the mean sdLDL-C was found to be elevated in the soft plaque group (39.46 ± 13.63 mg/dL) when compared to the calcific plaque group (35.41 ± 13.05 mg/dL), which was statistically significant (p<0.05) (Table 4). A cross-sectional study by Gupta et al. found that symptomatic patients with carotid artery disease had larger soft plaque on CT angiogram than asymptomatic patients, who mostly had calcific plaque [29]. Thus, elevated sdLDL-C, which has been associated with soft plaques, may be useful in predicting the risk of the development of symptomatic carotid artery disease. However, further prospective studies are needed to analyze its predictive capability.

There appears to be a shift in the outlook for determining the severity of atherosclerotic carotid disease. Plaque vulnerability, which indicates the risk for vascular events in the short term, is being considered for assessing the severity of the disease over the traditional way of using luminal narrowing [15]. In that way, soft plaque on CT angiogram, which is one of the key characteristics of vulnerable plaque, was associated with high sdLDL-C levels in this study.

One of the shortcomings of this study is its small sample size, with participants from a single tertiary health center. This study, being a retrospective cross-sectional study, could only establish association and not causation. Thus, future prospective studies involving a larger population from different centers should be conducted. The effect of chronic inflammatory disorders such as psoriasis, rheumatoid arthritis, and lupus on plaque formation and progression and their effect on sdLDL-C haven't been considered in this study.

## Conclusions

sdLDL-C, a more atherogenic subclass of LDL-C, is associated with atherosclerotic carotid plaque in this study. Patients with soft plaque on CT angiogram have significantly higher levels of sdLDL-C when compared to calcific plaques. Thus, it can be used as a simple and cost-efficient tool to assess carotid plaque vulnerability. It can also be employed as an adjunct along with LDL-C for monitoring hypolipidemic treatment, as it can easily be calculated with the existing lipid parameters that are commonly measured.
